# Postoperative seizure outcome and timing interval to start antiepileptic drug withdrawal: A retrospective observational study of non-neoplastic drug resistant epilepsy

**DOI:** 10.1038/s41598-018-31092-3

**Published:** 2018-09-13

**Authors:** Le Zhang, Xin-Yue Jiang, Dong Zhou, Heng Zhang, Shi-Min Bao, Jin-Mei Li

**Affiliations:** 1 Department of Neurology, West China Hospital, Sichuan University, Chengdu, Sichuan Province China; 2 Department of Neurosurgery, West China Hospital, Sichuan University, Chengdu, Sichuan Province China; 3 Department of Neurology, Hospital of Chengdu Office of People’s Government of Tibetan Autonomous Region, Chengdu, Sichuan Province China

## Abstract

This study aimed to investigate the impact of timing interval to start AED withdraw (TIW) after surgery on the seizure outcome in non-neoplastic drug resistant epilepsy (DRE). TIW were divided into three groups (respectively,<1 year, 1-<2 years, and ≥2 years). The seizure outcome at the different time points after starting AED withdrawal were compared among three groups. Other factors that related to seizure recurrence and TIW were included into the multiple analysis to investigate the predictors of seizure-free. Altogether, 205 patients were involved in the study. 102 individuals (50%) had seizure recurrence and 127 (62%) had seizure-free at the final follow up. 115 of them have attempted AED reduction and had not seizure recurrence before AED reduction. The rate of seizure-free had no significant difference among people with different TIW. Multiple analysis indicated that temporal surgery is a favorable predictor of seizure-free at the first year after starting AED withdrawal, and preoperative secondary generalized seizures is an unfavorable predictor of seizure-free at the final follow up. In patients with non-neoplastic DRE, TIW is not the mainly influence factor on seizure outcome, however, preoperative secondary generalized seizures and extra-temporal surgery are negatively associated with seizure-free.

## Introduction

Surgery is an effective treatment for patients with drug resistant epilepsy (DRE), and previous studies indicated that among people with poorly controlled epilepsy, patients who underwent surgery often had better control of seizure than those with medical therapy^1–4^. In these studies, surgery was proved to be of great benefit to patients with DRE either seizure freedom or quality of life.

In order to reduce the risk of seizure recurrence, patient with DRE frequently were recommended to continue to take AED until seizure free for two years after surgery^5^. However, the ideal outcome of surgery for epilepsy is to discontinue antiepileptic drugs (AED) without any seizure attack^6^. As early AED withdrawal at no cost of seizure outcome not only reduce the financial burden of patients but also is beneficial to neurodevelopment^7,8^, some other researchers put forward to start AED reduction before 2 years after surgery with completed seizure control^9^. Nevertheless, as for optimum timing to taper AED after surgery, survey of epileptologists showed that the duration for AED treatment after surgery were wildly different, ranging from less than one year to more than two years and no standardized guidelines exist^10–12^. How to find the suitable time point of AED withdrawal is difficult and controversial.

In previous studies, results about the time of AED withdrawal and seizure outcome are conflict. Some studies indicated that early reduction of AED increase the risk of relapse and is associated to lower rate of seizure free^13,14^, however, other studies showed that early AED withdrawal was not associated with seizure recurrence and had no affection on long-term seizure outcome^15–17^. Therefore, time point of withdrawing AED is a controversial but meaningful issue. However, most of previous studies included individuals with various pathological result, including tumor, which often had poorer outcome than those with non-tumor-associated epilepsy^18^, and few study specially focused on non-neoplastic DRE. This study aimed to assess the association between different time point of starting AED withdrawal and seizure outcome after epilepsy surgery in patients with non-neoplastic DRE.

## Materials and Methods

### Patients

We reviewed consecutive patients who underwent epilepsy surgery in West China Hospital form January 2010 to December 2014 and selected those patients according to the following criteria. The inclusion criteria included: (1) patients who conformed to the diagnosis of drug resistant epilepsy before surgery, defined as failure of adequate trials of two tolerated and appropriately chosen and used AED schedules (whether as monotherapies or in combination) to achieve sustained seizure freedom^19^; (2) patients whose pathological result presented non-neoplastic lesions; (3) patients who were followed up at least two years after surgery. The exclusion criteria included: (1) patients with previous epilepsy resectional surgery history; (2) patients who underwent hemispherectomy. Demographic characters, previous history, preoperative seizure type, MRI findings, surgery information, pathological results and medical strategies were collected from our database. Additional data, including adjusting strategy of AED and seizure frequency after surgery were obtained through telephone contact and outpatient clinic visits. And patients who had tried to reduce or discontinue AED after surgery were selected for further analyses about timing interval to start AED withdrawal (TIW) or timing interval to discontinue AED (TID) and seizure outcome, except patients who had postoperative seizure recurrence before AED reduction and who reduce or discontinue AED on their owns, even though seizure haven’t been controlled.

### Standard protocol, approval and consent

The study was approved by the Ethical Committee of West China Hospital, Sichuan University, on human experimentation protocols. Written informed consent was obtained from patient or their direct relatives. All methods were performed in accordance with the relevant guidelines and regulations.

### Measurement of AED and Seizure Outcome

Tapering AED were performed according to patients’ intention and physicians’ suggestion. The daily drug load was calculated as truly daily dose/defined daily dose (DDD). The DDD reflects the ‘assumed average maintenance dose per day for a drug used for its main indication in adults’ (http://www.whocc.no/atc_ddd_index/). The daily drug load was compared with the baseline (during surgery). A decrease of at least 10% was considered to be drug reduction, an increase of at least 10% was considered to be dose added, and minor changes between −10% and +10% were regarded as unchanged^17,20^. And if one kind of AED was replaced by another AED due to adverse side effect or poor efficacy, we didn’t regard it as reduction. TIW was divided as less than 1 year (<1 year), at least 1 year but less than 2 years (1-<2 years), and at least 2 years (≥2 years). Seizure outcome was identified according to International League Against Epilepsy (ILAE) classification^21^ and was evaluated at the first, second and third year after starting AED withdrawal and at the last follow up. Seizure-free was defined as having not epileptic seizure (ILAE class 1) at least one year with or without AED before the follow-up time. Seizure recurrence was defined as epileptic seizure reoccur again after surgery.

### Statistical analysis

Statistical analyses were performed using SPSS version 21.0. The rate of seizure-free among individuals with different TIW and TID is analyzed by Chi-squared test or Fisher’s exact test. To identify which factors are possibly related to seizure recurrence, univariate analyses are also performed in gender, age of onset, age at surgery, duration of epilepsy, location of surgery, side of surgery, preoperative seizure type, previous history, MRI results, number of AED immediately after surgery, and pathological results among individuals with seizure recurrence and those without. Age of onset, age at surgery, and duration of epilepsy are analyzed as continuous variables, number of AED immediately after surgery is analyzed as ranked data, and others are analyzed as categorical variables. The Wilcoxon test is used for continuous variables and ranked data, and the Chi-squared test is used for categorical variables. Factors that have significant difference between people with and without seizure recurrence in univariate analyses and TIW are included into multiple logistic regression models of seizure-free at the first, second and third year after starting AED withdrawal and at the final follow up. P values < 0.05 (two-sided) are considered to be significant.

## Result

**Figure 1:**
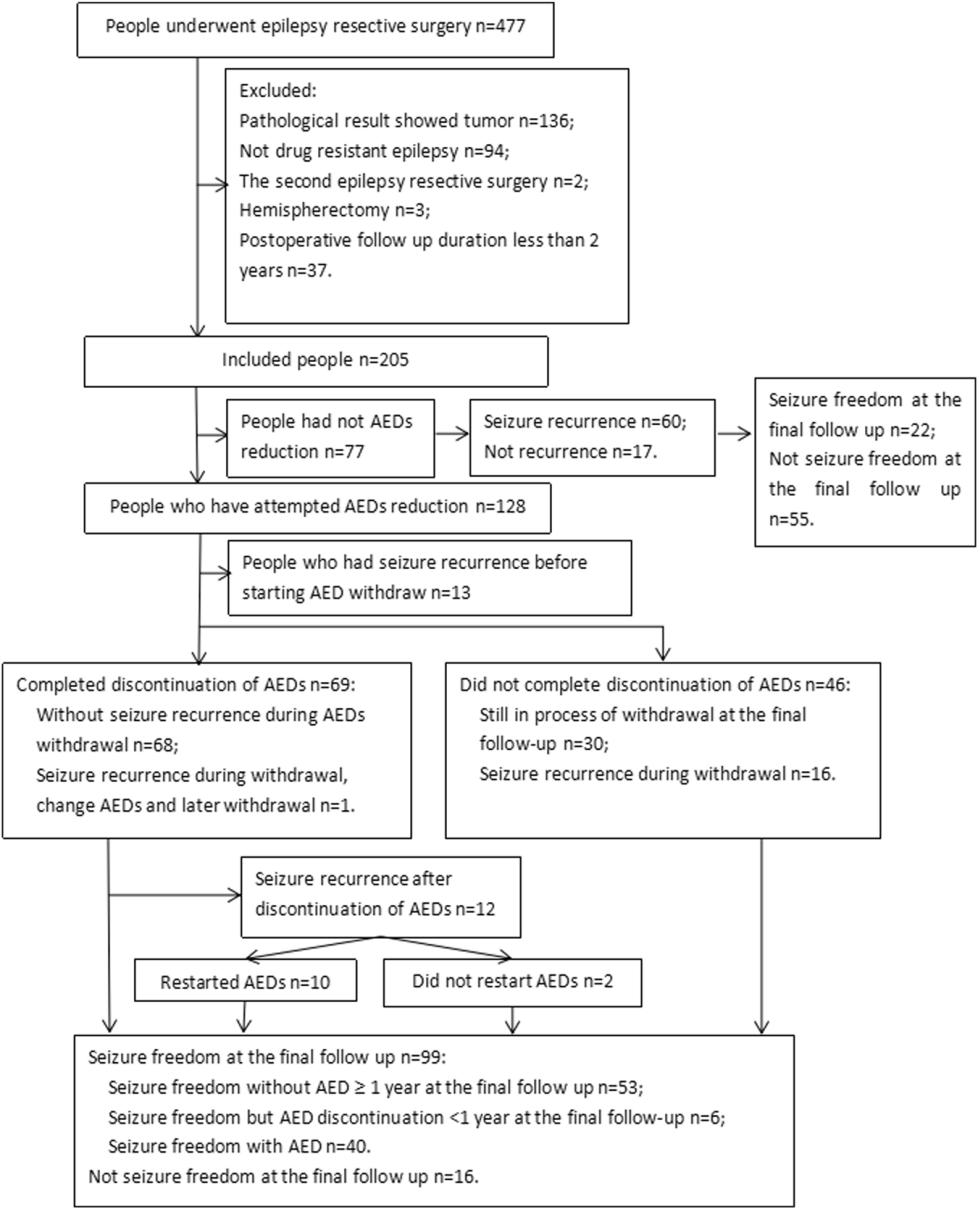
Flow diagram of people selection, the pattern of AEDs withdrawal and seizure outcome after surgery.

From January 2010 to December 2014, a total of 477 people underwent surgery in West China Hospital and 205 of them with a 54 months median follow up duration (inter-quartile range [IQR] 39–64.5 months, range from 24 to 80 months) were included in this study according to our include and exclude criteria (Fig. 1).

Median age at onset of epilepsy was 15 years old (IQR 9–20.5 years, range from 3 months to 60 years), median duration of epilepsy was 9 years (IQR 4–15 years, range from 0.5 to 32 years), and median age at surgery was 24 years old (IQR 19–33.5 years, range from 4 to 63 years). 127 patients (62%) underwent temporal surgery and 78 patients (38%) received extra-temporal surgery including 39 frontal, 9 parietal, 4 occipital, 16 in border area of two or three lobes and 10 had multiple lobes surgery. Before surgery, 55 patients (27%) only had focal seizures without secondary generalized seizures. And 34 patients (17%) had febrile convulsion history, 35 patients (17%) had brain trauma history, 23 patients had other disease history, including birth hypoxia, encephalitis and meningitis. 177 patients (86%) had abnormal MRI results. The pathological results of 89 people showed hippocampal sclerosis, 34 people showed vascular malformation, 33 people showed glial scar, and 49 people showed other results including focal cortical dysplasia, gliosis, evidence of inflammation, and normal. At the final follow up, 127 people (62%) had seizure-free.

### Factors that are related to seizure recurrence

During the follow-up duration, a total of 102 people (50%) had seizure recurrence during the follow up, the recurrent rate in 1 month after surgery is 17%, and with 24%, 30%, and 39% respectively in 6 months, 1 year and 2 years. Univariate analyses indicated that people with longer duration of epilepsy before surgery, extra-temporal surgery, and with secondary generalized seizures before surgery had higher rate of seizure recurrence after surgery (Table 1).

**Table 1:** Comparisons between individuals with and without seizure recurrence.

Variables	Recurrence n = 102 (50%)	No-recurrence n = 103 (50%)	P value
Onset age, years, median (IQR)	14.00 (9.00–20.00)	15.00 (7.00–22.00)	0.905^c^
Surgery age, years, median (IQR)	23.00 (19.00–34.00)	24.00 (17.00–33.00)	0.362^c^
Duration of epilepsy, years, median (IQR)	10.00 (5.00–15.00)	7.00 (4.0–13.00)	0.012^c^
Gender (female)	40 (39%)	46 (45%)	0.430^d^
Location of surgery			<0.001^d^
Temporal	49 (48%)	78 (76%)	
Extra-temporal	53 (52%)	25 (24%)	
Side of surgery (left)	59 (58%)	52 (50%)	0.290^d^
Preoperative seizure type			0.020^d^
Focal	20 (20%)	35 (34%)	
Secondary generalized	82 (80%)	68 (66%)	
Previous history			0.076^d^
No	50 (49%)	63 (61%)	
Febrile convulsion	15 (15%)	19 (18%)	
Trauma	21 (21%)	14 (14%)	
Others^a^	16 (16%)	7 (7%)	
Number of AED immediately after surgery			0.878^c^
1	11 (11%)	10 (10%)	
2	84 (82%)	88 (85%)	
≥3	7 (7%)	5 (5%)	
Abnormal MRI	88 (86%)	89 (86%)	0.978^d^
Pathology			0.209^d^
Hippocampal sclerosis	38 (37%)	51 (49%)	
Vascular malformation	17 (17%)	17 (17%)	
Glial scar	21 (21%)	12 (12%)	
Others^b^	26 (25%)	23 22%)	

Abbreviations: AED, antiepileptic drug; IQR, inter-quartile range.

^a^Others including birth hypoxia, encephalitis and meningitis;

^b^Others including focal cortical dysplasia, gliosis, evidence of inflammation, and normal;

^c^Wilcoxon test;

^d^Chi-squared test.

### Timing interval to start AED withdrawal and seizure outcome

Seventy-seven people did not reduce AED during follow up, including 32 increase the AED and 45 unchanged; 13 people have seizure recurrence before starting withdrawal of AED; and the pattern of AED withdrawal and seizure outcome for 115 people who had not seizure recurrence before reduction of AED was shown in Fig. 1.

**Figure 2:**
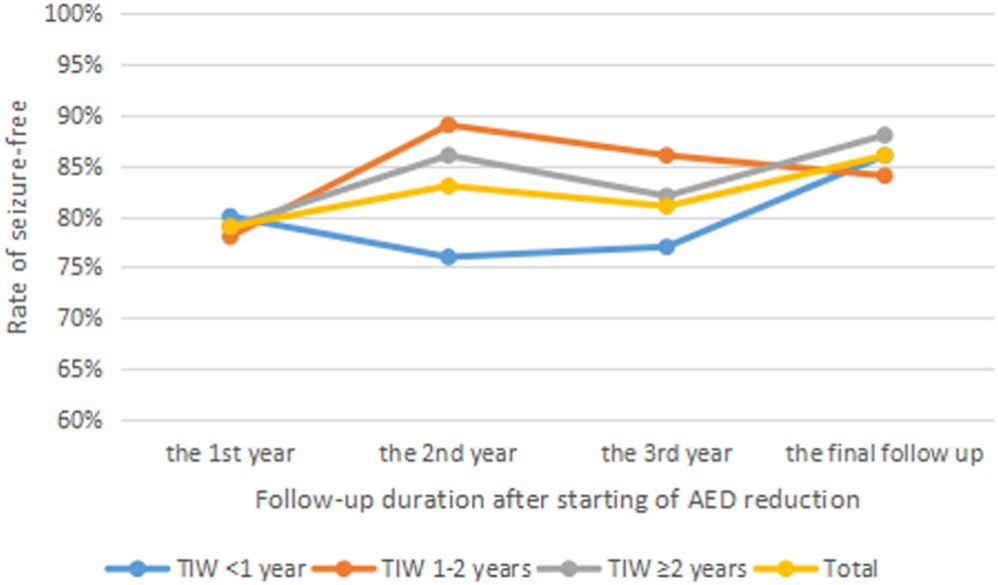
Rate of seizure-free at different follow up point among individuals with different TIW. No significant difference was found among three groups with different TIW at different follow up point (Chi-squared test or Fisher’s exact test, p > 0.05). Abbreviations: AED, antiepileptic drug; TIW, timing interval to start antiepileptic drug withdrawal.

Thirty-five people have started reduction of AED during less than one year after surgery, 32 people during one to two years, and 48 people maintain the dosage of AED at least two years after surgery. Median TIW is 18 months (IQR 9–24 months, range from 1 to 52 months). With a median follow up duration of 54 months (IQR 39–65 months, range from 24 to 79 months) after surgery and 36 months (IQR 15–48 months, range from 0.5 to 73 months) after start of AED withdrawal, 29 of 115 people (25%) had seizure recurrence during AED withdrawal or after AED discontinuation and 13 of 29 (45%) recurrent individuals regain seizure-free at the final follow up. 109 people had a more than one year follow up duration after starting of AED reduction, 80 people with more than two years and 62 people with more than three years. The rate of seizure-free at the first, second and third year after starting of AED withdrawal was 79%, 83% and 81%, respectively. At the final follow up, 99 individuals (86%) had seizure-free. As shown in Fig. 2A, the rate of seizure-free had no significant difference between groups with different TIW at the first or second or third year after starting of AED reduction or at the final follow up (p = 0.980, 0.429, 0.840, and 0.941, respectively).

TIW and factors that related to seizure recurrence, which identified by univariate analysis (p < 0.05), were included into multiple logistic regression analysis (Table 2). As individuals with shorter TIW tended to had longer follow-up duration after start of AED discontinuation (p < 0.001, median follow-up duration is 54, 39, and 16.5 months for individuals with TIW <1 year, 1-<2 years, and ≥2 years, respectively), follow-up duration after start of AED discontinuation was also included into the multiple analysis at the final follow up. Still none significant association was found between TIW and seizure-free at the first or second or third year after starting of AED reduction or at the last follow up. However, temporal surgery is a favorable predictor of seizure-free at the first year after starting AED reduction. Moreover, with secondary generalized seizures before surgery is an unfavorable predictor of seizure-free at the final follow up. 109 people had a more than one year follow up duration after starting of AED reduction, 80 people with more than two years and 62 people with more than three years.

**Table 2:** Multiple logistic regression analysis of factors that were possibly related to seizure free.

Variables	The first year (n = 109)			The second year (n = 80)			The third year (n = 62)			The final follow up (n = 115)		
	OR	95% CI	P value	OR	95% CI	P value	OR	95% CI	P value	OR	95% CI	P value
TIW	0.887	0.492–1.600	0.691	1.252	0.549–2.857	0.593	1.149	0.447–2.950	0.773	0.975	0.378–2.514	0.958
Duration of epilepsy	0.999	0.927–1.078	0.986	0.965	0.874–1.066	0.483	0.923	0.820–1.040	0.189	1.045	0.955–1.143	0.342
Location of surgery (temporal)	3.456	1.248–9.570	0.017	3.130	0.793–12.350	0.103	3.272	0.701–15.268	0.131	0.992	0.295–3.335	0.990
Preoperative seizure type (secondary generalized seizures)	0.388	0.113–1.333	0.133	0.163	0.019–1.392	0.097	0.230	0.025–2.096	0.192	0.105	0.013–0.856	0.035
Follow-up duration after start of AED discontinuation	—	—	—	—	—	—				1.002	0.960–1.046	0.925

Abbreviations: AED, antiepileptic drug; CI, confidence interval; OR, odds ratio; TIW, timing interval to start antiepileptic drug withdrawal.

### Timing interval to start AED withdrawal, timing interval to discontinue AED and seizure outcome in people who completed AED discontinuation

Sixty-nine of 115 individuals (60%) completed discontinuation of AED, with 10 people discontinue AED during less than one year after surgery, 11 people during one to two years, and 48 people did not discontinue AED until at least two years after surgery. Among these people, median TIW is 12 months (IQR 7.5–24 months, range from 1 to 45 months) and median TID is 24 months (IQR 18–36 months, range from 1 to 66 months). With a median follow up duration of 61 months (IQR 46–69.5 months, range from 24 to 79 months) after surgery, 40 months (IQR 28–56 months, range from 6 to 73 months) after start of AED withdrawal, and 28 months (IQR 13.5–42.5 months, range from 5 to 72 months) after complete discontinuation of AED, one people had seizure recurrence during withdrawal and reached seizure-free without AED at the final follow up and other 12 people had seizure recurrence after AED discontinuation. 6 of 12 people regain seizure-free at the final follow up, including 2 of them without AED and 4 of them with AED. 68 and 62 people had a more than one year, 59 and 43 people with more than two years, and 48 and 29 people with more than three years follow up duration respectively, after start AED withdrawal and after AED discontinuation. The rate of seizure-free at the first, second and third year after start AED withdrawal was 88%, 90% and 90%, respectively, and the rate of seizure-free at the first, second and third year after AED discontinuation was 85%, 91% and 93%, respectively. Additionally, the rate of seizure-free without AED at the first, second and third year after AED discontinuation was 85%, 84% and 90%, respectively. At the final follow up, 63 individuals (91%) had seizure-free, and 59 of them without AED.

**Figure 3:**
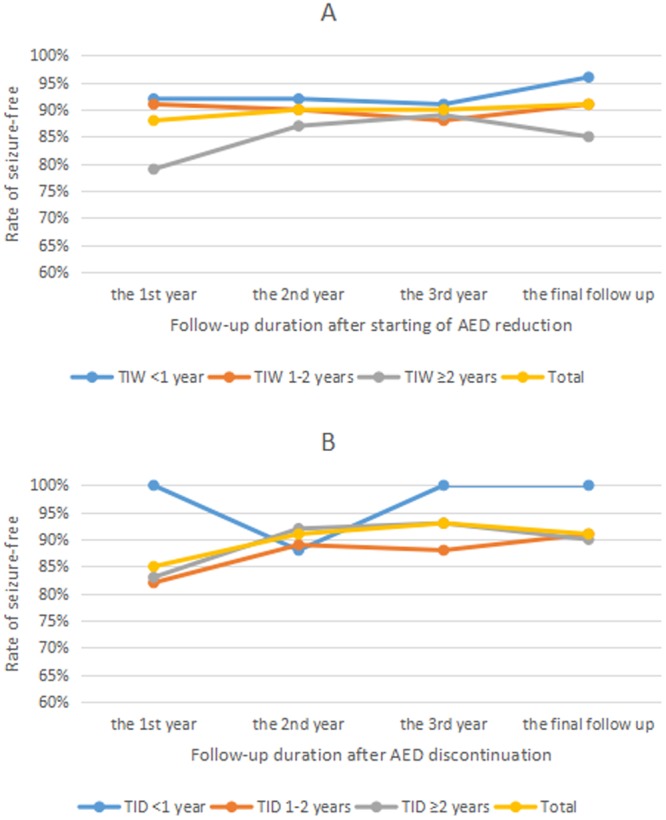
Rate of seizure-free at different follow up point among individuals who completed AED discontinuation. Neither significant difference was found among three groups with different TIW (**A**) nor with different TID (**B**) at different follow up point (Chi-squared test or Fisher’s exact test, p > 0.05). Abbreviations: AED, antiepileptic drug; TID, timing interval to discontinue AED; TIW, timing interval to start antiepileptic drug withdrawal.

The rate of seizure-free had significant difference between groups with different TIW at the first or second or third year after start AED withdrawal at the final follow up (p = 0.405, 0.872, and 1.000, respectively) (Fig. 3A). As shown in Fig. 3B, the rate of seizure-free had no significant difference between groups with different TIW at the first or second or third year after AED discontinuation at the final follow up (p = 0.555, 1.000, 1.000, and 0.821, respectively). Due to the limited sample size of people who completed AED discontinuation and there are less than 10 people in group without seizure-free at each follow up time point, multiple logistic regression analysis was not conducted among these people.

## Discussion

In this study, we have retrospective reviewed the surgery outcome of 205 individuals. Approximate 60% people had attempted to withdraw AED with seizure control, and among these, 60% completed AED discontinuation. And at the final follow up, 62% patients had seizure-free. The seizure outcome after surgery was similar to previous studies about epilepsy surgery^14,22^ and was obviously better than tumor associated epilepsy with about 34 percent of seizure-free^18^.

During the follow up, 50% individuals had seizure recurrence after surgery and nearly one third of them initial relapsed at the first month after surgery, which indicates the first month after surgery is the period of high risk recurrence, and patients should be more carefully taken care of during this period. In addition, previous studies indicated that delayed seizure-free or acute postoperative seizures were related to unfavorable seizure outcome after surgery^18,22–24^. What’s more, time interval to reach seizure-free after surgery was also reported to be associated with timing interval of start AED reduction^15,25^. Therefore, in this study, to avoid bias, we only included people without seizure recurrence before AED reduction (immediate seizure-free) for analysis about TIW and seizure outcome. And to these favorable people, early withdraw of unnecessary AED with no influence on seizure outcome is likely more feasible and beneficial than those with unfavorable outcome.

Time to withdraw AED after surgery is still controversial. Although, AED was advised to maintain for at least 2 years after surgery by some researchers^5^, some other researchers put forward that there were no added benefit to delay discontinuation of AED beyond 1 to 2 years of those with completed seizure control after surgery^9^ and starting reduction of AED at least 1 year after surgery was proved to be safe in some studies^9,22,26^. In this study, nearly two thirds of people (72%) start of AED withdrawal at least 1 year after surgery and nearly half of people (44%) start of AED withdrawal at least 2 years after surgery; and median TIW was 18 months. About two thirds of people (69%) discontinue AED at least 2 years after surgery and only 14% individuals discontinue AED less than 1 year after surgery; and median timing to discontinue AED was 24 months. Our result indicated that in west china, time to reduce and discontinue AED was similar to surveys from Canada and US^10,11^. And 25% individuals had seizure recurrence during or after AED reduction or discontinuation, which was also accord with previous studies of general epilepsy surgery population without age limited reported for 22–32% recurrent rate^22,24^, but was higher than studies of paediatric epilepsy with 12% recurrent rate^15^.

In this study, the statistical analysis showed that neither TIW had significant influence on seizure-free at the first, second and third year after starting AED withdrawal nor at the final follow up. Further analysis of people who completed AED discontinuation also indicated that neither TIW nor TID had influence on seizure outcome after surgery. Previous studies which focused on temporal lobe epilepsy also presented similar finding that postoperative adjustment of AED dosage had not impact on seizure outcome^17^. Therefore, we speculate that in patient with non-neoplastic DRE without seizure recurrence before AED reduction, TIW or TID is not a critical factor of short term seizure outcome (in three years). However, as the median follow-up duration after starting AED withdrawal was only 36 months in this study, the influence of TIW on long term seizure outcome (more than three years) is unknown and further studies are warranted.

Nevertheless, we found that people who had preoperative secondary generalized seizures had higher rate to seizure recurrence, and multiple analysis also identified it as an unfavorable predictor of seizure-free at the final follow up. Compared to only with focal seizures, those with secondary generalized seizures were likely more severity before surgery, which cause the poorer surgery outcome. Additionally, our study also showed that temporal surgery is an independent favorable predictor of seizure-free after AED reduction. Previous studies also indicated that temporal surgery had significantly better outcome than extra-temporal surgery^27,28^. Therefore, to people with preoperative secondary generalized seizures and underwent extra-temporal surgery, clinicians should be more cautious about AED withdrawal and more assessment should be carried.

Although, our studies provided some clues for further studies, we also have some limited. Firstly, mainly due to study design bias and the limitations inherent in uncontrolled retrospective research, people were not randomization, although adjust have been done, some bias still cannot be avoided and prospective randomized study are needed; Secondly, due to the relative shorter follow-up duration after the reduction of AED, the influence of TIW on long term seizure outcome need further study; Thirdly, we did not assess the influence of timing interval to start AED withdrawal on cognitive outcome and quality of life; Moreover, as only people without recurrence before reduction were included into analysis about TIW and seizure outcome, our findings cannot be generalized to other settings.

## Conclusion

Our study found that timing interval to start AED withdrawal and timing interval to discontinue AED after surgery is not related to seizure outcome in non-neoplastic drug resistant epilepsy. However, extra-temporal surgery and preoperative secondary generalized seizures are unfavorable predictors seizure-free after surgery. Therefore, it is likely other factors, rather than time to withdraw AED decide the seizure outcome after surgery. However, due to the limitation of retrospective design, small sample, and shorter follow-up duration, our outcome should be interpreted and extended with caution, and further prospective studies with larger sample urgently needed for this meaningful and valuable issue.
